# The New Era of Treatment for Obesity and Metabolic Disorders: Evidence and Expectations for Gut Microbiome Transplantation

**DOI:** 10.3389/fcimb.2016.00015

**Published:** 2016-02-19

**Authors:** Thilini N. Jayasinghe, Valentina Chiavaroli, David J. Holland, Wayne S. Cutfield, Justin M. O'Sullivan

**Affiliations:** ^1^Liggins Institute, The University of AucklandAuckland, New Zealand; ^2^Department of Infectious Diseases, Counties Manukau HealthAuckland, New Zealand; ^3^Gravida: National Centre for Growth and DevelopmentAuckland, New Zealand

**Keywords:** gut microbiome transplantation, microbiome, microbiota, obesity, treatment

## Abstract

**Key Points**

- The microbiome has been implicated in the development of obesity.
- Conventional therapeutic methods have limited effectiveness for the treatment of obesity and prevention of related complications.
- Gut microbiome transplantation may represent an alternative and effective therapy for the treatment of obesity.

Obesity has reached epidemic proportions. Despite a better understanding of the underlying pathophysiology and growing treatment options, a significant proportion of obese patients do not respond to treatment. Recently, microbes residing in the human gastrointestinal tract have been found to act as an “endocrine” organ, whose composition and functionality may contribute to the development of obesity. Therefore, fecal/gut microbiome transplantation (GMT), which involves the transfer of feces from a healthy donor to a recipient, is increasingly drawing attention as a potential treatment for obesity. Currently the evidence for GMT effectiveness in the treatment of obesity is preliminary. Here, we summarize benefits, procedures, and issues associated with GMT, with a special focus on obesity.

## Introduction

Obesity has recently been identified as a disease by the American Medical Association with >33% of the world's adult population (20 years and older) overweight or obese (World Health Organization, 2014). Sadly, this is projected to increase to the point where up to 57.8% of the world's population aged 20 and over is either overweight or obese (World Health Organization, 2014). There are various causative factors that contribute to the development of obesity including genetics (Wang et al., 2011a), low levels of physical activity and exercise, poor diet and other unhealthy behaviors. Obesity is a major risk factor for diabetes, hypertension, and metabolic syndrome. Despite the promotion of numerous strategies for the prevention and treatment of obesity, most patients are refractory to treatment. Thus, new approaches are currently being sought to reduce the financial, social, and health consequences of the obesity epidemic.

The human gut contains an extensive population of microbes (the gut microbiome) that effectively constitute a microbial “endocrine organ” (Cani and Delzenne, 2007; Clarke et al., 2014). Recent research has implicated these microbes as having a significant role in the development of obesity (Bäckhed et al., 2004; Ley et al., 2005, 2006b; Turnbaugh et al., 2006, 2009; Backhed et al., 2007; Zhang et al., 2009), diabetes (Larsen et al., 2010; Qin et al., 2012), and cardiovascular disease (Ordovas and Mooser, 2006; Wang et al., 2011b; Howitt and Garrett, 2012; Tang and Hazen, 2014). Therefore, environmental effects on these microbes and our ability to manipulate them in a controlled manner are under increasing scrutiny.

Fecal/gut microbiome transplantation (GMT; Box 1) has been suggested as a new method of altering the gut microbiota that may lead to beneficial metabolic changes (Smits et al., 2013). Modifications of the host's microflora by GMT were first performed in the 1950s to treat pseudomembranous colitis now known to be due to *Clostridium difficile* infection (CDI) (Eiseman et al., 1958). Since then, GMT has been successfully used for CDI treatment and is increasingly considered the treatment of choice for chronic pseudomembranous colitis (Gough et al., 2011). Despite the fact that GMT has been shown to improve insulin sensitivity in adults with features of metabolic syndrome (Vrieze et al., 2012), its application as a therapy for other conditions, including obesity, is still experimental. As such, it is still unclear how, when, or under which circumstances GMT should be performed. Here, we will address the procedures, benefits, and issues associated with GMT, with a special focus on obesity.

Box 1 **FMT vs. GMT.**

Through-out this manuscript we refer to gut microbiome transplantation (GMT) and not fecal microbiome transplantation (FMT). Predominant amongst our reasons for this minor change in terminology is the public attitude and perception of products and treatments derived from feces as being “dirty” or “unhygienic” (Brandt, 2012; Leslie et al., 2014). These prejudices are ingrained and continually reinforced by the testing and notifications of fecal contamination of public drinking and bathing sources that form part of a public system to identify and prevent disease outbreaks. Moreover, the eating of feces (i.e., coprophagia) is recognized as a symptom of mental health disorders (Zeitlin and Polivy, 1995). Collectively, these conscious and sub-conscious prejudices combine to reduce the potential acceptability of fecal transfers. Therefore, in order for microbiome transfer to be implemented as a widespread treatment for chronic and non-acute disorders, it must be promoted in a way that minimizes the fecal stigma. We propose that the first step in this journey is the use of the term GMT.

## The human host

A human being is more than the sum of their “own cells.” Rather, the ~10 trillion human cells that we each contain constitute < 10% of the cells within our bodies with the remaining ~100 trillion cells, that reside in and on the human body, being of microbial origin (Ley et al., 2006a). As a consequence of this, our ~20,000 human genes (Yang et al., 2009) are vastly outnumbered by the human microbiome's 2 to 20 million microbial genes (at least 100 times the number of human genes; Knight, 1993). These microbial genes (99%) are mostly encoded by the bacteria within the human gut (Qin et al., 2010). It is now becoming increasingly clear that these microbial communities interact with the human host at many levels, which include the local and systemic gut and immune function (Macpherson and Harris, 2004).

The microbes comprising the human microbiome generally have a symbiotic relationship with the host. The human intestine provides them with a supply of nutrition and a relatively stable living environment. In return, microbes play a vital role in our body by synthesizing metabolites (e.g., vitamin K, thiamine, biotin, folic acid, vitamin B_12_; Gorbach, 1996), digesting non-starch polysaccharides into additional nutrients for the human host (Vercellotti et al., 1977), providing a physical barrier in the form of a biofilm to boost the immune system, and protecting from pathogens (Mazmanian et al., 2005). Moreover, intestinal microbes may be also an important factor for brain development (Diaz Heijtz et al., 2011), metabolic function, and hormones and neurochemicals production (Lyte, 2013).

## The development of human gut microbiome

The human gut is generally considered to be sterile *in utero* (Ley et al., 2006a; Maynard et al., 2012), with microbial colonization beginning during delivery when newborns come into contact with maternal womb, vaginal, fecal, and skin microbes (Lee and Polin, 2003). However, meconium of healthy neonates, collected within 2 h of delivery from healthy mothers, has been shown to contain microbes (e.g., *E. fecalis, S. epidermidis*, and *E. coli*; Jiménez et al., 2008). This has led to the promotion of hypotheses that bacteria from the maternal gut are transferred to amniotic fluid, possibly via the circulation (Kornman and Loesche, 1980), and through swallowing of amniotic fluid into the fetal gut (Goldenberg et al., 2008; Neu and Rushing, 2011). Given that a fetus swallows 400–500 ml of amniotic fluid per day late in gestation (Goldenberg et al., 2008; Neu and Rushing, 2011), only low numbers of microbes would be required within the amniotic fluid to facilitate microbial colonization of the fetal gut. This mechanism of fetal colonization is supported by the detection of microbes and microbial products within amniotic fluid isolated from healthy mothers (Li et al., 2014). Finally, microbes have been isolated from the umbilical cord (Jiménez et al., 2005) and placenta (Aagaard et al., 2014) of healthy infants (without infection or inflammation). Collectively these observations are consistent with the hypothesis that fetus is colonized by microbes before birth.

Mode of delivery (e.g., vaginal delivery or cesarean section) has been observed to have a significant impact on the microbiota within the newborn gut (Dominguez-Bello et al., 2010; Neu and Rushing, 2011). Interestingly, children born by cesarean section have a greater risk of obesity in later childhood, suggesting a causal link between early gut bacterial colonization and later obesity (Blustein and Liu, 2015). Cesarean section has been associated with a greater likelihood of *C. difficile* and lower number of *Bacteroides* spp. colonization (Penders et al., 2005, 2006). Gestational age of newborns (e.g., were they born prematurely, at term or post-term) also correlates with gut microflora composition. The gut of preterm infants contains higher levels of *C. difficile* compared to full term infants (Penders et al., 2006). Moreover, data obtained from short-term stool culture have shown that colonization by *Bifidobacterium* and *Lactobacillus* is delayed in preterm infants, whereas colonization by potentially pathogenic bacteria (especially *E. coli*) is increased (Westerbeek et al., 2006; Butel et al., 2007).

During infancy, diet is one of the many contributors to the development of gut microbiome (Koenig et al., 2011). The importance of diet is reinforced by observations that breast-fed infants have more *Bifidobacteria* than formula-fed infants (Koenig et al., 2011). By contrast, formula-fed infants have a lower microbial density, yet higher diversity of other microbial species compared to breast-fed infants (Harmsen et al., 2000; Koenig et al., 2011). After the introduction of solid food into the diet, at weaning, an adult-like microbial ecology begins to develop within the gut (Fanaro et al., 2007).

By 3–4 years of age, the gut microbiome composition is dominated by two phyla (>90% of bacteria): *Firmicutes*, which are pro-inflammatory and obesogenic, and *Bacteroidetes*, which protect from these effects (Cani and Delzenne, 2007; Clarke et al., 2014). Once established, the gut microbiota remains relatively stable throughout the life of healthy adults albeit subject to temporary modifications (Palmer et al., 2007). There are two broad groups of influences on the gut microbiome: dynamic factors (diet and drugs) and less dynamic factors (genetic, early events/exposures, and lifestyle factors). Diet contributes to dynamic changes in gut microbiome and influences approximately half of the microbial population activity (Zhang et al., 2010). Conversely, other factors tend to maintain the activity of the microbial population. However, microbial composition undergoes changes in the elderly (Tiihonen et al., 2010), which include increases in the levels of *Lactobacilli, Coliforms, Clostridium*, and *Enterococci* and a decrease in the number of *Bifidobacterium* (Mitsuoka, 1990). The presence of imbalance in the composition of the gut microbiota at all ages, which is also known as “dysbiosis,” is associated with obesity development (Bäckhed et al., 2004; Ley et al., 2005; Turnbaugh et al., 2009).

In otherwise healthy individuals, diet quality is the major modulator of the gut microbiota, accounting for 57% of host gut bacterial variation (Zhang et al., 2010). Diet-induced changes to gut microbial content are relatively rapid, occurring over 3–4 days and are readily reversible (Walker et al., 2011). Modification of gut microbiome can also be achieved by use of prebiotics and probiotics, and antibiotics (Walker et al., 2011; Binns, 2013; Modi et al., 2014). Prebiotics and probiotics appear to support a more favorable gut environment (Binns, 2013). However, these supplements need to be consumed regularly to maintain changes in gut microbiota (Binns, 2013), as it is unclear how long these changes last in the gut. Short- and long-term modifications of gut microbiome can also result from antibiotics, which reduce diversity by promoting the elimination of some bacterial species and antibiotic resistance by horizontal transfer within the remaining flora (Modi et al., 2014). Alcohol consumption also affects composition of gut microflora (Mutlu et al., 2012), with chronic alcohol consumption causing microbial dysbiosis, a reduction in the number of *Bacteroidetes* and an increase in the numbers of *Proteobacteria* present in the gut (Mutlu et al., 2012). Alterations in gut microbiome in alcoholic subjects correlate with increased levels of serum pro-inflammatory toxins (Mutlu et al., 2012). However, a recent study on microbiome development showed that microbial metabolites and their metabolic pathways are constant from birth, although microbial diversity increases with age and becomes more consistent from the age of 3 years (Kostic et al., 2015).

## Composition of human microbiome

Each individual has their own unique microbial population whose composition is affected by host genetic make-up, history of exposure to microbes, age, diet, environment, and geographical location (The Human Microbiome Project Consortium, 2012; Ursell et al., 2012; Yatsunenko et al., 2012). Moreover, even within an individual there are a myriad of distinct environments each of which is colonized by different microorganisms (e.g., skin, oral cavity, gastrointestinal, respiratory, and urogenital tracts; Gerritsen et al., 2011). It is universally accepted that the high surface area and availability of nutrients make the gut an ideal site for microbial growth (Gebbers and Laissue, 1989; Sekirov et al., 2010). However, the gut microbiota composition changes at different sites within the gut (Zoetendal et al., 2002) and even within the different layers of the gut epithelium (Swidsinski et al., 2005). Despite this complexity, the ease of collection and the high microbial content (Hütter et al., 2012) mean that fecal matter is generally used to study “the gut microbiome.” Therefore, despite the fact that the numbers of bacteria are several orders of magnitude larger in the distal colon, which seems to have a relatively uniform composition of microbes (Whitman et al., 1998; Eckburg et al., 2005; Ley et al., 2006a; Gerritsen et al., 2011), this does not reflect the situation throughout the entire gut. As such, it must be borne in mind that fecal bacteria do not necessarily inform on the composition of the microbiome within the distinct environments that are present throughout the gut and are characterized by differing levels of pH, oxygen levels, and food transit rates.

## How do we characterize the microbiota?

The application of metagenomic techniques (Kim et al., 2013) to the study of the composition, functional capacity, ecology, and integration of human microbiota with human cellular metabolism (Tremaroli and Bäckhed, 2012) is increasing our knowledge of how this “microbial organ” integrates into the human system. Metagenomic techniques overcome limitations of conventional bacterial cell culture and other molecular techniques that have been applied to the study of the gut microbiome (Table 1; Aslam et al., 2010). The Human Microbiome Consortium, the European Consortium of the Meta-HIT and the International Human Microbiome Consortium are currently developing and applying these techniques to understand microbial effects on human health and diseases (Kim et al., 2013).

**Table 1:** **Techniques used for the analysis of microbial communities**.

**Category**	**Advantages**	**Disadvantages**
Denaturing gradient gel electrophoresis (DGGE)	• A comparative tool for the study of inter-sample microbial composition. Useful for studying microbial population changes over a specific time period (Vaughan et al., 2000). • Specific taxonomic information can be obtained by band extraction followed by re-amplification and sequencing (Heuer et al., 1999; Riemann and Winding, 2001). • Less expensive than other techniques.	• Bias due to PCR (von Wintzingerode et al., 1997) and different DNA extraction rates (Theron and Cloete, 2000). Provides a semi-quantitative measure of species abundance (Vaughan et al., 2000). • Limited by cultivation techniques, especially for strict anaerobes (Vaughan et al., 2000). • The ecological role of microbes cannot be determined (Heuer et al., 1999).
16S amplicon sequencing	• Culture independent technique (Rajilić-Stojanović et al., 2007) used to detect a wide range of microbes by amplification and sequencing of variable regions within the16S rRNA sequence (Vaughan et al., 2000).	• PCR bias (Sipos et al., 2010; Schloss et al., 2011). • On its own, it does not inform on microbes functionality within samples (Vaughan et al., 2000).
Metagenomics, metatranscriptomics, and metaproteomics	• Culture independent techniques that identify gene composition and functional outputs of the microbes present in a sample (Verberkmoes et al., 2009). • Ideally performed as a combination of metagenomics (populations' DNA complement), metatranscriptomics (population's RNA composition), and metaproteomics (population's protein composition) (Verberkmoes et al., 2009).	• Expensive (Wooley and Ye, 2009). • Complex bioinformatics (Meyer et al., 2008). • Extraction biases.

Conventional techniques for the identification and characterization of microbial communities are mostly culture dependent and are unable to easily identify all of the microorganisms present and functional contributions that specific microorganisms make to the complex biological environments in which they exist (Verberkmoes et al., 2009). Despite their expense, metagenomic studies overcome many of these limitations.

## Connecting the gut microbiome to obesity and cardio-metabolic disorders

Four bacterial phyla (i.e., *Firmicutes, Bacteroidetes, Proteobacteria*, and *Actionobacteria*) account for the majority of the bacteria present in the human gut (Khanna and Tosh, 2014). Typically ~60% of the bacteria present in the human gut belong to the gram positive *Bacteroidetes* or gram negative *Firmicutes* phyla (Bäckhed et al., 2005). The most commonly found gut bacteria genera in adults are *Bifidobacterium, Lactobacillus, Bacteroides, Clostridium, Escherichia, Streptococcus*, and *Ruminococcus* (Conlon and Bird, 2015). Individually and collectively, these bacteria produce a vast range of microbial products that include enzymes for carbohydrate metabolism (Xu et al., 2003), short chain fatty acids (SCFA) (Bergman, 1990), lipopolysaccharide (LPS) (Munford, 2008), and secondary bile acids (Nicholson et al., 2012). These microbial products can enter into the human circulation where they contribute to energy flux in the human, or cause inflammation and other complications (Tehrani et al., 2012; Trompette et al., 2014).

The gut microbial composition is distinctive in obese individuals, and tends to show reduced complexity (Turnbaugh et al., 2009). For example, obese mice have reduced numbers of *Bacteroidetes* and increased numbers of *Firmicutes* when compared to lean mice (Ley et al., 2005). These changes in gut microbial populations have significant implications for energy homeostasis, as a 20% increase in *Firmicutes* and a corresponding 20% decrease in *Bacteroidetes* is estimated to provide an additional 150 kcal of energy per day to an adult human (Jumpertz et al., 2011). *Lactobacillus* numbers have also been observed to increase in obese people, while anorexic patients show higher numbers of *Methanobrevibacter smithii* (Armougom et al., 2009).

Early research into the relationship between the gut microbiome and obesity has used 16S ribosomal RNA (rRNA) gene sequences to examine microbial diversity in obese and lean individuals. Numerous studies have found phylum-wide differences in lean or obese individuals (Eckburg et al., 2005; Ley et al., 2006b; Frank et al., 2007). However, findings on the relative proportions of the main phyla in obese and lean individuals are contradictory (Duncan et al., 2008; Turnbaugh et al., 2009; Schwiertz et al., 2010; Bervoets et al., 2013; Colson et al., 2013; Ferrer et al., 2013). Meta-analysis has shown that the microbial changes associated with obesity are not simply phylum based but are the result of a collection of numerous small differences within the overall population structure (Walters et al., 2014). Therefore, it is important to look at the overall composition of the gut microbial population structure as an indicator of obesity rather than simply the proportion of *Bacteroidetes* to *Firmicutes*.

Type 2 diabetes has also been linked with gut microbiota that differ from that found in a healthy individual (Larsen et al., 2010; Qin et al., 2012). Patients with type 2 diabetes have reduced level of butyrate-producing bacteria and more pathogenic bacteria (Qin et al., 2012). These patients also show more *Betaproteobacteria* and reduced *Firmicutes* and *Clostridia* levels compared to healthy subjects (Larsen et al., 2010). Furthermore, a correlation has been observed between *Bacteroidetes* to *Firmicutes* ratio and plasma glucose concentration in type 2 diabetic and obese patients (Larsen et al., 2010; Schwiertz et al., 2010). With these observations, it is clear that manipulating the microbiome composition may represent a novel approach for preventing and treating obesity and related alterations.

Several recent in-depth reviews provide detailed information about potential mechanisms through which the microbiome is linked to the development of obesity (Hartstra et al., 2014; Gérard, 2015). The association between characterization of an altered gut microbiome in obese or diabetic subjects does not demonstrate cause and effect. However, there are indications that the gut microbiome actively contributes to the development of obesity. Specifically, Backhed et al. compared the fat mass of germ-free and conventionally raised mice, and showed that intestinal microbes are able to control fat storage (Bäckhed et al., 2004). Similarly, Turnbaugh et al. introduced an “obesogenic microbiota” to germ-free mice and found that mice with obesogenic microbes developed more body fat than those with “lean microbiota” (Turnbaugh et al., 2006).

Various mechanisms have been proffered to explain the association of an “obese microbiota” with higher fat content in mice (Bäckhed et al., 2004; Ley et al., 2005; Turnbaugh et al., 2006; Hartstra et al., 2014). Most simply, microbial mediated degradation of dietary fiber to SCFA contributes additional calories to the host (Bäckhed et al., 2004; Hartstra et al., 2014). In addition, SCFAs, notably butyrate, facilitates enhanced insulin sensitivity and fatty acid oxidation in muscle and reduced hepatic lipogenesis as well as increased satiety (Hartstra et al., 2014). The way in which butyrate leads to these changes is unclear, however it is likely to involve the activation of the G protein coupled receptors GPR41 and GPR43, which are involved in glucose metabolism (Hartstra et al., 2014). Moreover, SCFA and bacterial lipopolysaccharides activate Toll-Like receptor 4 (TLR4) and signal intracellular inflammatory pathways related to the induction of insulin resistance and increased adiposity (Tsukumo et al., 2007; Tehrani et al., 2012).

Turnbaugh et al. observed a higher content of SCFAs (e.g., butyrate and acetate) in the large intestine of obese mice (Turnbaugh et al., 2006) consistent with a mechanism that involves increased absorption of SCFA (Bäckhed et al., 2004). In addition, comparisons of normal mice on a high-fat diet with germ free mice on the same diet have demonstrated that the gut microbes can reduce the expression of host fasting-induced adipose factor/angiopoietin-like protein-4, a lipoprotein lipase inhibitor (Ley et al., 2005). Reduced expression of fasting-induced adipose factor increases lipoprotein lipase activity and triglycerides storage in hepatic cells (Bäckhed et al., 2004), again contributing to alterations to patterns and levels of fat deposits. Despite these potential mechanisms, the exact contribution(s) that changes in the proportions of *Firmicutes* to *Bacteroides* species make to the development of obesity remains unknown (Ley et al., 2006b). More work is required to more accurately understand the contributions of the many proposed mechanisms linking the gut microbiome with obesity, particularly in humans.

## Can we manipulate the microbiome to prevent and treat obesity and its related complications?

Lifestyle modifications are an important part of obesity management. However, lifestyle interventions (such as diet and exercise) have not consistently led to appreciable weight loss (Golan et al., 1998). Furthermore, pharmacotherapy may have negative impacts on the physiology and psychology of obese patients (Collins, 1988; Hill et al., 1994; Hill, 2007). Surgical interventions (e.g., Bariatric surgery) can be effective for short term-to-medium-term weight management in severely obese patients (Gloy et al., 2013). However, there are significant risks associated with surgical interventions [e.g., dumping syndrome (rapid gastric empting), micronutrient malabsorption, cholelithiasis, and hypoglycaemia] (Puzziferri et al., 2014; Tack and Deloose, 2014) and the treatment is expensive (Encinosa et al., 2005). Therefore, new approaches for the prevention and treatment of obesity are required. GMT represents an excellent and economic (Encinosa et al., 2005) option for individuals who are unable to lose weight by lifestyle measures, or those who cannot undergo surgical treatment.

As gut microbes have been implicated in the development of obesity (Turnbaugh et al., 2006), replacement of a microbial population (“bad” microbes) that promotes obesity with a population that promotes a healthy state (“good” microbes) may represent a possible treatment. The question remains: how do you change the entire flora of an individual at once? GMT with fecal bacteria transferred from unaffected individuals to affected recipients has been suggested as a promising method of altering and improving gastrointestinal microbiota and human health (Aroniadis and Brandt, 2013; Smits et al., 2013).

GMT uses live microorganisms as a potential intervention that “confers a beneficial health effect on the host.” Thus, the fecal samples can be considered a probiotic (Park and Bae, 2015). However, unlike typical probiotics, GMT doesn't modify the recipient's gut flora using microorganisms associated with fermentation. Instead, GMT modifies the recipient's gut flora using a community of organisms that was isolated from a healthy gut—that is the same biological niche. This approach is essential for the modification of the gut flora in obesity because of the multiplicity of small, yet predictive, differences between the flora of obese and lean individuals (Walters et al., 2014).

GMT is not new. In the fourth century A.D., Chinese patients suffering from severe diarrhea were given oral fecal suspensions (Zhang et al., 2012). Likewise, in the sixteenth century stool was used to treat diarrhea, fever, vomiting and constipation (Zhang et al., 2012). In modern times (1958), fecal enemas have been used as a cure of human pseudo-membranous colitis (Eiseman et al., 1958). The use of GMT as a treatment for any disease, except recurrent *C. difficile* (CD) infection, requires an approved investigational new drug (IND) permit according to the US Food and Drug Administration (FDA) (Moore et al., 2014). As such, most studies of the effects of GMT on obesity have been limited to mice (Table 2).

**Table 2:** **Mice studies on gut microbiome transplantation**.

**Mouse model**	**Treatment**	**Outcome**	**References**
Adult germ-free C57BL/6 mice	Colonized with normal microbiota harvested from cecum of adult conventionally raised mice and fed on low fat-polysaccharide-rich diet.	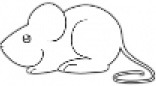 Increase in body fat content (60%) and insulin resistance despite reduced food intake.	Bäckhed et al., 2004
Adult germ-free C57BL/6J mice	Transplantation of microbes taken from the caecum of: -obese (ob/ob) mice with greater relative abundance of Firmicutes.	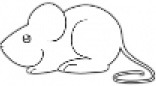 Increase in relative abundance of Firmicutes and body fat.	Turnbaugh et al., 2008
	-lean (+/+) donors with a smaller relative abundance of Firmicutes.	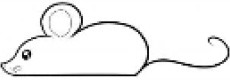 Decrease in relative abundance of Firmicutes and body fat.	
Adult germ-free C57BL/6J mice	Transplanted germ free mice with fecal microbiota from adult human female twin pairs; discordant for obesity and those mice were fed on low-fat, high polysaccharide diet.	Mice transplanted with microbiota from an obese twin developed higher adiposity than mice with the microbiota from a lean twin.	Ridaura et al., 2013

*
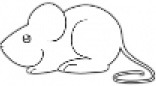
 Obese mice. 
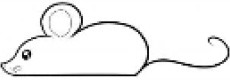
Lean mice*.

We contend that, when considering the potential efficacy of the GMT approach for obesity, it is more appropriate to reflect on the meta-analyses of the effectiveness of fecal transfers in the treatment of *C. difficile* and inflammatory bowel disease (Kassam et al., 2013; Colman and Rubin, 2014). Until recently, there was no consistently effective treatment for recurrent *C. difficile* infection, which leads to considerable morbidity, including chronic diarrhea, colitis, and toxic megacolon, as well as a reported mortality of up to 38% (Hota et al., 2012). However, GMT is being increasingly viewed as the treatment of choice for recurrent *C. difficile* infection. Moreover, meta-analyses of clinical trials have consistently demonstrated that gut microbiome transfer is efficacious and safe [IBD, pooled cure rate 36.2% (95% CI 17.4–60.4%); *C. difficile*, pooled cure rate 89.1% (95% CI 84–93%)] (Kassam et al., 2013; Colman and Rubin, 2014). Finally, a recent study in patients with *C. difficile* colitis has shown that gut microbiome transfer causes a significant shift in composition from the diseased state to one equivalent to that seen for healthy individuals by the human microbiome project (Weingarden et al., 2015). As such, gut microbiome transfer holds significant promise as a treatment for the rapid and concerted modification of an unhealthy flora.

GMT is now being considered for a wider range of disorders, including severe obesity and type 2 diabetes mellitus. To date, investigation of the therapeutic benefit of GMT in adult obesity or type 2 diabetes has been limited to a single pilot study. Vrieze et al. performed a short-term GMT study in nine treated and nine control middle-aged men with metabolic syndrome (Vrieze et al., 2012), with transfer via a naso-duodenal tube. Six weeks after GMT, treated subjects had an impressive 75% increase in insulin sensitivity. Furthermore, GMT was associated with favorable changes to gut microbiota that included greater bacterial diversity and a 2.5-fold increase in butyrate-producing bacteria (Vrieze et al., 2012). However, the study was not continued long enough to evaluate the full potential of therapy, notably on body weight, and composition.

Whilst gut microbiome transfer in humans offers so much promise, it is not clear yet whether it actually leads to significant weight loss. Moreover, the duration of the effect, treatment composition, and mode of delivery required to achieve optimum weight loss must be established. There are currently 17 clinical trials registered (USA, Europe, and Australia) to test the efficacy of GMT as a clinical treatment, mostly for *C. difficile* infection. Only two of these trials are looking at GMT as a means of treating obesity. However, the reverse effect (lean to obese) has been demonstrated as the result of use of an overweight donor for the treatment of recurrent *C. difficile* infection (Alang and Kelly, 2015). It remains clear that there are considerable practical and safety issues that need to be considered and overcome before GMT can be used as a routine clinical or non-clinical intervention (Box 2).

Box 2 **Practical and safety issues of GMT.**

- **Choice of donor** (Andrews et al., 1995; Jakobsson et al., 2010; Bakken et al., 2011; Pérez-Cobas et al., 2013; Viaud et al., 2013; Kostic et al., 2014; Panda et al., 2014)
  ∘ Related, unrelated or universal? There is debate over the relative merits of using related or unrelated donors (Bakken et al., 2011).
  ∘ Once chosen, donors must be screened for: conditions associated with microbial dysbiosis (e.g., metabolic syndrome, morbid obesity, chronic fatigue syndrome, inflammatory bowel syndrome, irritable bowel syndrome, chronic diarrhea or constipation, GI malignancy, CD toxins); intestinal pathogens (e.g., Giardia, *Cryptosporidium, Isopora* and Rotavirus, Hepatitis A, B and C, HIV, Syphilis, and *Helicobater pylori*); antibiotic use within the previous 3 months; immunosuppressive treatments and anti-cancer agents; high risk-sexual behaviors; illegal drug use; recent travel to areas with endemic diarrhea, or recent body piercings/tattoos.
- **Donor feces preparation** (Berg et al., 1988; Lund-Tønnesen et al., 1998; Persky and Brandt, 2000; Mueller et al., 2006; Kostic et al., 2014)
  ∘ The use of fresh or frozen feces.
  ∘ It is unclear if the solvent (saline, non-bacteriostatic milk, yoghurt, or water), method of homogenization (hand stirring, shaking, or blender), or filtration (coffee filter, gauze pad, or steel strainer) make a difference to transfer efficiency (Persky and Brandt, 2000; Borody et al., 2015).
  ∘ There is currently no recommended standardized amount of feces suggested for use in GMT.
- **Route of administration and site of inoculation** (Lund-Tønnesen et al., 1998; Mueller et al., 2006; Yang et al., 2009; Silverman et al., 2010; Borody and Khoruts, 2012; Kostic et al., 2014)
  ∘ Retention enemas/naso-gastric tube/naso-jejunal tube/upper tract endoscopy (esophagogastroduodenoscopy)/colonoscopy/self-administered enemas.

## What are the challenges associated with GMT

**Figure 1:**
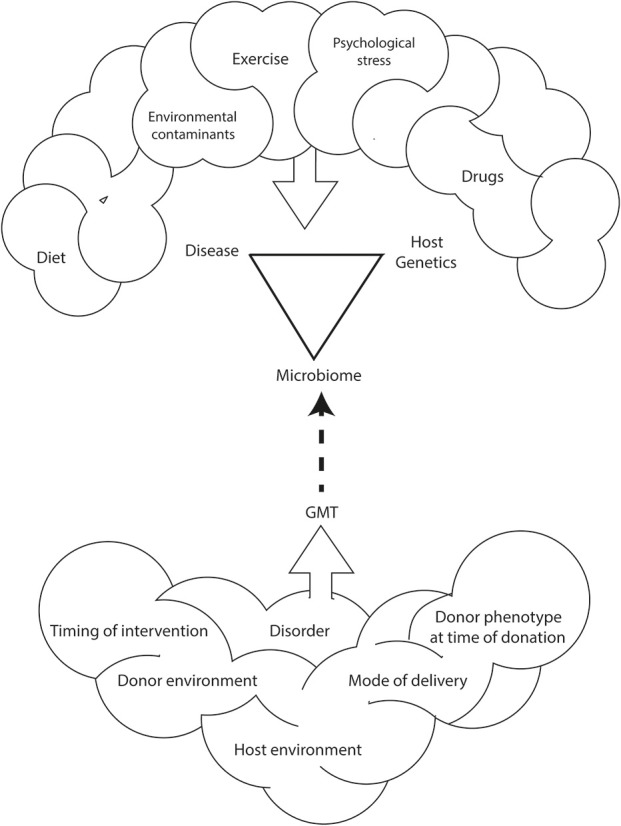
**Environmental and genetic interactions between the host and the host's microbiome impact the development and incidence of obesity and related disorders**. This relationship is also affected by diet, exercise, psychological stress, and environmental contaminants. As such, methods for human microbiome manipulation, including GMT, may represent a revolutionary approach for the treatment of non-communicable diseases including obesity.

GMT is a promising treatment for antibiotic resistant *C. difficile* infection. However, the use of GMT as a treatment for metabolic diseases such as obesity or type 2 diabetes is only experimental (Bäckhed et al., 2004; Turnbaugh et al., 2008; Vrieze et al., 2012; Ridaura et al., 2013). There is still much to be learnt about the GMT method that includes: characteristics of the ideal donor, delivery formulation (e.g., in solution, encapsulation), mode of administration (e.g., oral, nasojejunal, or rectal), duration of benefit and, thus, frequency of treatment (Figure 1).

Limited data suggests that GMT is a safe treatment (Borody and Khoruts, 2012; Vrieze et al., 2012; Van Nood et al., 2013) that has not currently been found to be associated with the development of new infections or diseases (Brandt et al., 2012). Therefore, further studies are required to monitor the long-term side-effects of GMT on both donors and recipients. These studies should also test the theoretical and practical benefits and side-effects of using fecal transplants as a treatment for obesity. These include: (1) the cost, ease of intervention, and relative safety of the non-invasive GMT as opposed to gastric by-pass surgery and pharmaceutical interventions; (2) the chances that GMT causes non-specific short- and long-term side-effects similar to those caused by pharmaceutical interventions; and (3) the psychological stress associated with the procedure (e.g., effects of performance anxiety on the donor, Brandt, 2013).

The psychological stresses and social stigma associated with feces mean that some patients find GMT to be an unappealing treatment (Zipursky et al., 2012). However, a survey of CDI patients found that regardless of GMT's unappealing nature, patients are willing to try it (Zipursky et al., 2012). Whether this willingness to try GMT as a treatment would translate to obese patients is yet to be determined. However, if GMT is shown to be an effective treatment for obesity then there will inevitably be greater refinement of the transplanted microbiota into a more palatable and optimally efficacious formulation.

## Conclusion

Changes in the ratio of different gut microbial species have been associated with onset and development of several disorders, including obesity (Ley et al., 2005). It can be assumed that gut microbiota impacts on host metabolism through the promotion of increased uptake of monosaccharides, storage of triglyceride, digestion of dietary fiber (Bäckhed et al., 2004), and synthesis of hormonal precursors (Hartstra et al., 2014). Use of GMT to treat several disorders (e.g., chronic *C. difficile* infection) has already been established. However, it remains to be determined if GMT may be successful also for other diseases, such as obesity and its related complications. Based on the available evidence, GMT may represent a novel and successful intervention that could potentially transform the management of severe obesity in children and adults. Randomized controlled trials are required to confirm outcomes, efficacy and long-term safety of GMT in the treatment of obesity. The role of specific bacteria/species and combinations of intestinal microbiota should be clearly addressed beyond simply the change in body fat, ideally through longitudinal analysis of the meta-genomic, -proteomic, and -transcriptomic composition of donor and recipient's gut microbial content, before and after GMT. This characterization of GMT effects must include determining whether the process simply changes the composition of the existing microbial population or if it results in the complete transplantation of a non-obese microbial population. In conclusion, GMT represents a very real and potentially revolutionary treatment for obesity.

## Author contributions

TJ wrote the manuscript. VC contributed to the writing of the manuscript. DH commented on the manuscript. WC and JO conceived, directed, and contributed to the writing of the manuscript.

### Conflict of interest statement

The authors declare that the research was conducted in the absence of any commercial or financial relationships that could be construed as a potential conflict of interest.
